# Overexpression of the MYB29 transcription factor affects aliphatic glucosinolate synthesis in *Brassica oleracea*

**DOI:** 10.1007/s11103-019-00890-2

**Published:** 2019-06-12

**Authors:** Diana L. Zuluaga, Neil S. Graham, Annett Klinder, A. E. Elaine van Ommen Kloeke, Angelo R. Marcotrigiano, Carol Wagstaff, Ruud Verkerk, Gabriella Sonnante, Mark G. M. Aarts

**Affiliations:** 1 Laboratory of Genetics, Wageningen University, Droevendaalsesteeg 1, 6708 PB Wageningen, The Netherlands; 2 Plant and Crop Sciences Division, School of Biosciences, University of Nottingham, Sutton Bonington Campus, Loughborough, LE12 5RD Leicestershire UK; 3 Department of Food and Nutritional Sciences, University of Reading, PO Box 226, Whiteknights, Reading, RG6 6AP UK; 4 Department of Ecological Science, Faculty of Earth and Life Sciences, VU University Amsterdam, De Boelelaan 1085, 1081 HV Amsterdam, The Netherlands; 5 Department of Soil, Plant and Food Science, University of Bari ˝Aldo Moro˝, Bari, Italy; 6 Food Quality and Design, Wageningen University, P.O. Box 17, 6700AA Wageningen, The Netherlands; 7 Institute of Biosciences and Bioresources, National Research Council, Via G. Amendola 165/A, 70126 Bari, Italy

**Keywords:** MYB29 transcription factor, Brassicaceae plants, Glucosinolates, Isothiocyanates, Sinigrin, Antigenotoxic effect

## Abstract

**Key message:**

Overexpression of BoMYB29 gene up-regulates the aliphatic glucosinolate pathway in *Brassica oleracea* plants increasing the production of the anti-cancer metabolite glucoraphanin, and the toxic and pungent sinigrin.

**Abstract:**

Isothiocyanates, the bio-active hydrolysis products of glucosinolates, naturally produced by several *Brassicaceae* species, play an important role in human health and agriculture. This study aims at correlating the content of aliphatic glucosinolates to the expression of genes involved in their synthesis in *Brassica oleracea*, and perform functional analysis of *BoMYB29* gene. To this purpose, three genotypes were used: a sprouting broccoli, a cabbage, and a wild genotype (Winspit), a high glucosinolate containing accession. Winspit showed the highest transcript level of *BoMYB28*, *BoMYB29* and *BoAOP2* genes, and *BoAOP2* expression was positively correlated with that of the two *MYB* genes. Further analyses of the aliphatic glucosinolates also showed a positive correlation between the expression of *BoAOP2* and the production of sinigrin and gluconapin in Winspit. The Winspit *BoMYB29* CDS was cloned and overexpressed in Winspit and in the DH AG1012 line. Overexpressing Winspit plants produced higher quantities of alkenyl glucosinolates, such as sinigrin. Conversely, the DH AG1012 transformants showed a higher production of methylsulphinylalkyl glucosinolates, including glucoraphanin, and, despite an up-regulation of the aliphatic glucosinolate genes, no increase in alkenyl glucosinolates. The latter may be explained by the absence of a functional *AOP2* gene in DH AG1012. Nevertheless, an extract of DH AG1012 lines overexpressing *BoMYB29* provided a chemoprotective effect on human colon cells. This work exemplifies how the genetic diversity of *B. oleracea* may be used by breeders to select for higher expression of transcription factors for glucosinolate biosynthesis to improve its natural, health-promoting properties.

**Electronic supplementary material:**

The online version of this article (10.1007/s11103-019-00890-2) contains supplementary material, which is available to authorized users.

## Introduction

*Brassica oleracea* (CC genome) is a member of the *Brassicaceae* family and one of the three diploid *Brassica* species in the “triangle of U” (Nagaharu 1935) that also includes the diploids *B. rapa* (AA) and *B. nigra* (BB) and the allotetraploids *B. juncea* (AABB), *B. napus* (AACC) and *B. carinata* (BBCC). Each of the *Brassica* genomes has undergone a specific whole-genome triplication (Lysak et al. 2005). *Brassica oleracea* comprises many important vegetable crops including cauliflower, broccoli, cabbages, Brussels sprouts, kohlrabi and kales, which are high in carotenoids (Kopsell and Kopsell 2006) and contain diverse glucosinolates (GSLs), secondary metabolites, naturally produced by plants of the Brassicaceae family (Traka and Mithen 2009). A group of GSL products derived from enzymatic hydrolysis, isothiocyanates (ITCs), play important roles in human health and agriculture. Isothiocyanates are considered the main components responsible for the cancer-preventing properties of *Brassica* plants upon consumption by humans (Traka and Mithen 2009). For instance, sulforaphane, the aliphatic ITC generated from hydrolysis of 4-methylsulphinylbutyl GSL (glucoraphanin), exhibits chemopreventive properties, prohibiting the development of cancer cells via suppression of cytochrome P450 enzymes, induction of apoptotic pathways, inhibition of angiogenesis, anti-inflammatory activity and suppression of cell cycle progression (Juge et al. 2007; Abdull Razis et al. 2017). Sulforaphane also activates the human Nrf2 transcription factor promoting the action of phase II detoxification enzymes, thus increasing cell defence against oxidative damage and enhancing the elimination of carcinogens (Chi et al. 2015; Su et al. 2018). Conversely, different types of ITCs, derived from aliphatic and aromatic GSLs, offer plant protection against several pathogens when used as a natural pesticide. For example, application of the aliphatic allyl ITC hydrolysis product of the 2-propenyl GSL (sinigrin) provides control of potato cyst nematodes (Pinto et al. 1998; Aires et al. 2009), soil-borne phytopathogenic fungi (Tiznado-Hernández and Troncoso-Rojas 2006), mealy cabbage aphid colonies (Newton et al. 2009), and beneficial soil invertebrates (Zuluaga et al. 2015). The natural toxic properties of ITCs make them useful as a more sustainable alternative to chemical fumigants in agricultural practices.

The aliphatic GSL biosynthetic pathway has been widely studied and described in *Arabidopsis thaliana*, including the identification of regulatory factors and structural genes involved in the process. Upon genome sequencing, putative orthologues of the *A. thaliana* genes have been found in *B. rapa* (Wang et al. 2011) and *B. oleracea* (Yi et al. 2015). For instance, *BoGSL*-*ALK*, the homolog of the *A. thaliana AtAOP2* gene, encodes a 2-oxoglutarate-dependent dioxygenase, essential for the conversion of methylsulfinylalkyl GSLs to their alkenyl forms, therefore being one of the major genes controlling the glucoraphanin content in *Brassica* crops. In *B. rapa* all three *BrAOP2* paralogues are active but have functionally diverged, *BrAOP2.1* showed a slightly different pattern of expression in below-ground tissue at the seedling stage and in the silique at the reproductive stage compared with *BrAOP2.2* and *BrAOP2.3* genes (Zhang et al. 2015). Heterologous expression and in vitro enzyme assays and Arabidopsis mutant complementation studies showed that all three *BrAOP2* genes encode functional *BrAOP2* proteins that convert the precursor methylsulfinyl alkyl glucosinolate to the alkenyl form (Zhang et al. 2015). *Brassica oleracea* var. *italica* (broccoli) is known to contain a high concentration of glucoraphanin compared to other *B. oleracea* varieties, whereas sinigrin is only present in negligible concentrations, or even absent (Rangkadilok et al. 2002). Li and Quiros (2003) found that the accumulation of methylsulfinylalkyl GSLs in broccoli is linked to the presence of a non-functional allele of *BoGSL*-*ALK* in this crop. Therefore, the allelic variation for this *BoAOP2* homolog is a key element for defining the potential health promoting effect of *B. oleracea* genotypes. The analysis of full-length cDNA libraries of kale, *B. oleracea* var. *acephala*, allowed to identify several genes related to the GSL biosynthetic pathway in this crop, including the transcription factor *BoMYB29*, the functional orthologue of the *A. thaliana MYB29* gene controlling the expression of GSL biosynthesis genes (Araki et al. 2013).

The hypothesis behind this work is that BoMYB29 is one of the key transcription factors that controls expression of AGSL biosynthesis genes; upregulation of this transcription factor may be obtained through ectopic overexpression, which can increase the expression of AGSL biosynthesis genes and consequently the concentrations of one or several AGSL, including those that are human or plant health promoting. To verify this hypothesis, we correlated AGLS content to the expression of genes involved in the synthesis of these compounds in wild and cultivated *B*. *oleracea* genotypes, and performed a functional analysis of the *BoMYB29* gene. Two genotypes showing a different AGLS profile were transformed with *BoMYB29* driven by the CaMV 35S promoter and transgenic plants were analysed for gene expression and AGLS production. The antigenotoxic activity of the *BoMYB29* overexpressing plants was evaluated by assessing the potential of plant extracts to protect human colon cancer HT29 cells from DNA damage. The results obtained in this work provide useful information for breeding programmes focused on improving health-promoting properties of *B. oleracea* or anti-herbivore traits.

## Materials and methods

### Plant material and growth conditions

*Brassica oleracea* seeds from the genotypes listed below were sown in 17-cm pots containing a peat-based commercial potting compost mixture (Lentse potgrond nr. 4; 85% peat, 15% clay). Plants grew in a conditioned greenhouse set at a 8 h/16 h dark/light photoperiod at 22 °C.

For expression experiments and HPLC analysis on non-transgenic material, three *B. oleracea* genotypes were used: Winspit (WIN); the purple sprouting broccoli (*B. oleracea* var. *italica* Plenck) F1 hybrid cv. ‘Santee’ (PSB); and the Savoy cabbage (*B. oleracea* var. *sabauda* L.) F1 hybrid cv. ‘Wintessa’ (SAV). Winspit, a natural wild accession collected close to Winspit in the United Kingdom (Gols et al. 2008), was provided by Rieta Gols (Wageningen University, the Netherlands) and had proved to contain high levels of GSLs compared to other wild and cultivated *B*. *oleracea* plants (Gols et al. 2008; Harvey et al. 2011; Zuluaga et al. 2015). The purple sprouting broccoli cv. ‘Santee’ and the Savoy cabbage cv. ‘Wintessa’ were provided by companies as cultivars with high GSL levels. Since WIN seeds were collected in the field and are expected to be genetically heterogeneous, leaf material from four individual WIN plants (WIN5, WIN7, WIN9 and WIN14) was collected and analysed separately, with three technical replicates for each biological sample. For both PSB and SAV, 12 plants each were considered, divided in four groups of three plants each and analysed per group, since plants were assumed to be genetically homogeneous. Three mature, fully expanded, but not old, leaves were harvested per plant, after approximately 8 weeks, snap frozen in liquid nitrogen and stored at – 80 °C until expression analysis was done.

For genetic transformation, Winspit and *B. oleracea* doubled haploid genotype DH AG1012 were used. DH AG1012 is derived from a cross between *B. oleracea* var. *alboglabra* (A12DHd) and *B. oleracea* var. *italica* (Green Duke GDDH33) (Sparrow et al. 2006). Seeds of DH AG1012 were provided by Penelope Sparrow (John Innes Centre, Norwich, UK).

### Cloning procedure and plasmid construction

The coding sequence (CDS) of the *B. oleracea MYB29* gene, paralogue 2 (*BoMYB29*) was isolated from the wild accession Winspit (Table S1). The *BoMYB29* cDNA was amplified by reverse transcriptase (RT-)PCR using the primers *BoMYB29_Gateway_F* and *BoMYB29_Gateway_R* and WIN7 leaf cDNA as a template. The 954 bp *BoMYB29*-*CDS* (GenBank MK522798) PCR product was agarose-gel-purified (Qiagen, Crawley, UK) and cloned into the pENTR™/D-TOPO Gateway donor vector (Invitrogen, http://www.invitrogen.com/) to create an entry clone. The insert was transferred from the entry clone into the pK7WG2D destination vector (VIB, Gent, Belgium) as described by the manufacturer (Invitrogen) to generate a 13,743-bp *Pro35S*::*BoMYB29*::*Ter35S* expression vector. This plasmid was transferred into *Agrobacterium tumefaciens* strain AGL1 (Lazo et al. 1991) by electroporation. This *A. tumefaciens* clone was used for DH AG1012 and Winspit transformation. AGL1 containing the pK7WG2D empty vector was used as a negative control for transformation (EV plants).

### *Brassica oleracea* transformation and regeneration

Transformation of the DH AG1012 and Winspit genotypes was carried out following the method of Sparrow et al. (2006) with minor modifications. The AGL1 *A. tumefaciens* strain was grown on LB-agar medium containing 100 mg/L carbenicillin and 100 mg*/*L spectinomycin for selection and incubated at 28 °C for 48 h. A single colony was transferred to 10 mL of LB medium containing the same selection antibiotics and grown while shaking at 28 °C for 48 h. A 50-µL aliquot of the bacterial suspension was used to inoculate a 10-mL LB liquid culture, without antibiotics, which was grown overnight at 28 °C in a shaker. DH AG1012 and Winspit seeds were surface-sterilized by immersion in 100% ethanol for 2 min and subsequently in a 15% (v/v) solution of sodium hypochlorite for 15 min, upon which seeds were finally rinsed three times in sterile distilled water. The seeds were germinated on full-strength MS (Murashige and Skoog 1962) medium plus vitamins, supplemented with 3% sucrose and 0.8% Phytoblend (Caisson Laboratories, USA) at pH 5.7. Cotyledons were used for *A. tumefaciens* transformation. After 72 h in co-cultivation, the cotyledons were transferred to selection medium (co-cultivation medium supplemented with 160 mg/L Timentin and 15 mg/L kanamycin as the selective antibiotics). Regenerating T_1_ green shoots were excised and transferred to Gamborg’s B5 medium consisting of Gamborg’s B5 salts (Gamborg et al. 1968), plus 1% sucrose, 0.8% Phytoblend, 160 mg/L Timentin and 25 mg/L kanamycin. After 3 weeks, T_1_ shoots were transferred to pots with 50 mL of Gamborg’s B5 medium with a higher concentration of kanamycin (50 mg/L). After transformants had developed a dense root system, T_1_ plants (primary transgenic plants) were transferred to sterile peat in pots as described in the’Plant material and growth conditions’ section. The transgenic nature of the plants was confirmed by a PCR analysis using the NPTII primers (Table S1). The expected sized fragment of 700 bp was obtained for all transgenic lines and was not present in WT plants.

Six independent *BoMYB29* T_1_ transgenic lines from DH AG1012 (DH1–DH6), and two primary T_1_ transgenic lines from Winspit (W1 and W2) were obtained. Transgene expression was analysed by qPCR in all the T_1_ lines and, as negative control, in three WT DH AG1012 and three Winspit plants. *BoGAPDH* was used as reference gene (Broekgaarden et al. 2008). T_2_ seeds, obtained by self-crossing of T_1_ plants, were collected from W1 and W2 and from the three DH AG1012 lines with the highest expression of the transgene (DH3, DH5, DH6), surface-sterilized and then grown on Gamborg’s B5 medium supplemented with 50 mg/L kanamycin. The germinated, kanamycin-resistant T_2_ transgenic seedlings were transplanted into soil. Six kanamycin-resistant T_2_ plants were selected per line (DH3, DH5, DH6, W1 and W2 lines), for transgene expression analysis (as described above). The three T_2_ transgenic plants of each line that showed the highest transgene expression values, were used for further expression and phenotypic analysis. Three mature fully expanded, but not old, leaves from three wild-type (WT) plants and three selected T_2_ transgenic plants from each *BoMYB29* line, as well as empty vector plants (EV), were harvested after 8 weeks, stored at – 80 °C and used for expression or/and phenotypic analysis.

### Microarray analysis

The Affymetrix Brassica Exon 1.0 ST Array (Love et al. 2010) was used for gene expression analysis (microarray analysis). Hybridizations were carried out at the Nottingham Arabidopsis Stock Centre Affymetrix service (NASC, University of Nottingham, UK). Total RNA was obtained from the Winspit plants WIN5, WIN7, WIN9, WIN14, the PSB and SAV plant pools and the transgenic and wild-type DH AG1012 plants DH3, DH5, DH6, W1, W2, using the RNeasy Mini Kit (Qiagen). Total RNA samples were labelled, hybridised, and scanned following the standard protocol from the manufacturer (GeneChip Expression Analysis, Affymetrix, www.affymetrix.com). The GeneChip Command Console Software (AGCC; Affymetrix) was used to generate ‘.cel’ files for each of the hybridisations. The raw chip data were normalised using the Robust Multichip Average (RMA) pre-normalisation algorithm (Irizarry et al. 2003) in the GeneSpring GX (version 11.5; Agilent Technologies) analysis software package. Following RMA pre-normalisation, the signals were further normalized by standardizing the signal value of each probe-set to the median of that probe-set across all hybridisations. All further analysis was carried out using different functions in GeneSpring GX software. Differentially expressed genes were identified when the WIN plants were analysed together using a two-step process; (i) an unpaired *T* test using a Benjamini–Hochberg false discovery rate correction (p < 0.05) and (ii) a fold-change > 1.5 (Benjamini and Hochberg 1995). When the WIN plants were analysed separately, a probe-set was identified to be differentially expressed if the fold-change was > 2. Gene ontology (GO) analysis was performed using the GO analysis function in Genespring GX, with the p-value calculated using a hypergeometric test with Benjamini-Yekutieli correction (Benjamini and Yekutieli 2001). The GO analysis was performed on the *A. thaliana* paralogue of the *Brassica* gene, identified as the top hit from a BLAST (using NCBI BLASTn algorithm) analysis of the probe sequences against the *A. thaliana* genome.

### PCR and real-time quantitative PCR

Real-time quantitative reverse transcriptase PCR (qRT-PCR) was used to investigate expression of selected *B. oleracea* AGSL biosynthetic genes. Gene expression was analysed using the SYBR Green dye and a CFX96™ Real-Time PCR Detection System (Bio-Rad Laboratories, Richmond, CA). Expression levels were calculated relative to the expression of the *BoGAPDH* reference gene. The different isoforms/paralogues of each *B. oleracea* GSL gene were distinguished using specific primers. Primers for gene expression analysis were designed based on *B. oleracea* sequences if available; otherwise, *B. rapa* sequences were used (Table S1). For DH AG1012 *BoGS*-*ALK* (*AOP2*), PCR and sequencing were carried out with the odd48 and odd12 primers used by Gao et al. (2004) for broccoli.

Gene sequences were retrieved from the *Brassica* (BRAD; http://brassicadb.org/brad/) and GenBank (http://www.ncbi.nlm.nih.gov/genbank/) databases. Most of the evaluated genes were chosen according to the gene inventory of the GSL pathway and *B. rapa* orthologues (Wang et al. 2011). Primers for expression analysis of *BoGSL*-*ELONG*, *BoGSL*-*ELONG*-*L*, *BoGSL*-*PRO* and *BoGSL*-*PRO*-*L* genes were designed based on literature information (Li and Quiros 2002; Gao et al. 2006). Total RNA was isolated using the RNeasy Mini Kit (Qiagen) and reverse transcribed into cDNA, using the Script cDNA Synthesis Kit (Bio-Rad Laboratories, Richmond, CA, USA) according to the manufacturer’s instructions. qRT-PCR experiments were carried out using 50 ng of cDNA and the iQ SYBR Green Supermix (Bio-Rad) following the manufacturer’s protocol. Relative quantification of each single gene expression was performed using the comparative C_T_ method as described in the ABI PRISM 7700 Sequence Detection System User Bulletin #2 (Applied Biosystems, Foster City, CA, USA). The relative expression levels were represented as heat maps, using BAR Heatmapper Plus software (http://bbc.botany.utoronto.ca/ntools/cgi-bin/ntools_heatmapper_plus.cgi). Correlation analysis between expression of AGSL pathway genes and AGSL content in the *B. oleracea* plants/genotypes was performed using the SPSS Statistics 17.0 Software (http://www.spss.com). Genes that were not expressed or expressed at very low levels were not included in the analysis. Pearson correlation analysis was performed between expression of AGSL genes and AGSL content in *B. oleracea* genotypes (Tables 1, S2a–d), and between expression of AGSL pathway genes and AGSL content in the *B. oleracea WinMYB29* overexpressors (Tables 2, S2e, f). Correlation value was considered statistically significant at “*” p < 0.05 and “**” p < 0.01.

**Table 1:** Pearson correlation analysis between expression of AGSL genes and AGSL content in *B. oleracea* wild genotypes Winspit (WIN5, WIN7, WIN9, WIN14), in the purple sprouting broccoli ‘Santee’, and in the Savoy cabbage ‘Wintessa’

Gene name	BCAT4		GSL-Elong	GSL-Elong-L	GSL-PRO	GSL-PRO-L	BCAT3		CYP79F1	CYP83A1	SUR1	UGT74B1	UGT74C1	
Paralogue	1	2					1	2	1	1	1		1	2
AGSL														
GIB	0.278	− 0.326	0.295	− 0.424	0.532	− 0.306	− 0.457	− 0.437	0.129	− 0.192	− 0.272	0.206	0.438	− 0.668*
SIN	− 0.138	− 0.067	0.520	− 0.129	0.026	0.007	− 0.005	0.012	0.787**	− 0.103	− 0.198	− 0.002	0.307	− 0.194
GNP	− 0.215	− 0.225	0.506	− 0.156	− 0.037	− 0.024	− 0.049	0.001	0.737**	− 0.029	− 0.115	0.186	0.336	− 0.236
GBN	0.823**	0.011	− 0.329	− 0.197	− 0.313	− 0.164	− 0.538	− 0.358	− 0.138	− 0.459	− 0.067	− 0.223	0.325	− 0.368
PRO	− 0.432	0.242	− 0.379	0.461	− 0.313	0.289	0.491	0.458	− 0.347	0.213	0.222	− 0.191	− 0.637*	0.723**
SIN + GNP	− 0.202	− 0.197	0.512	− 0.152	− 0.025	− 0.018	− 0.041	0.003	0.751**	− 0.043	− 0.132	0.152	0.333	− 0.230
Total	− 0.151	− 0.225	0.513	− 0.188	− 0.002	− 0.046	− 0.098	− 0.041	0.752**	− 0.082	− 0.157	0.154	0.370	− 0.290

Gene name	ST5b				FMOGS-OX		AOP2	GSL-OH	BZO1p	MYB28			MYB29	
Paralogue	1	3	4	5	2	5	2	2	2	1	2	3	1	2
AGSL														
GIB	−0.407	0.493	− 0.427	− 0.375	0.716**	− 0.417	0.123	− 0.395	− 0.418	0.056	− 0.216	− 0.334	0.111	0.096
SIN	0.112	0.883**	0.069	− 0.022	− 0.011	0.755**	0.771**	− 0.158	− 0.121	0.897**	0.215	0.656*	0.981**	0.967**
GNP	0.077	0.907**	0.037	− 0.056	0.005	0.736**	0.837**	− 0.193	− 0.147	0.858**	0.176	0.595*	0.974**	0.969**
GBN	− 0.297	− 0.079	− 0.197	− 0.134	0.424	− 0.526	− 0.520	− 0.098	− 0.205	− 0.120	0.409	− 0.606*	− 0.224	− 0.214
PRO	0.392	− 0.660*	0.408	0.369	− 0.764**	0.278	− 0.217	0.421	0.456	− 0.310	0.038	0.205	− 0.328	− 0.331
SIN + GNP	0.084	0.909**	0.043	− 0.050	0.002	0.745**	0.830**	− 0.188	− 0.143	0.872**	0.184	0.611*	0.982**	0.976**
Total	0.045	0.940**	0.008	− 0.080	0.063	0.697*	0.817**	− 0.218	− 0.179	0.870**	0.191	0.560	0.982**	0.974**

Numbers below gene names indicate gene paralogues

*GIB* 3-methylsulphinylpropyl, *SIN* 2-propenyl/Allyl, *GNP* but-3-enyl, *GBN* pent-4-enyl, *PRO* pent-4-enyl, (2R)-2-hydroxybut-3-enyl

*^,^**Correlation is significant at the 0.05 and 0.01 level, respectively (1-tailed)

**Table 2:** Pearson correlation analysis between expression of AGSL pathway genes and AGSL content in the *B. oleracea BoMYB29* overexpressors DH3, DH5, DH6, W1 and W2

Gene name	BCAT4		GSL-Elong	GSL-Elong-L	GSL-PRO	GSL-PRO-L	BCAT3		CYP79F1	CYP83A1		SUR1		UGT74B1
Paralogue	1	2					1	2	1	1	2	1	2	
AGSL														
GIB	0.903*	0.966**	0.970**	0.922*	0.712	0.880*	0.992**	− 0.190	0.963**	0.986**	0.757	0.975**	0.735	0.951*
GRA	0.784	0.874	0.906*	0.835	0.861	0.885*	0.985**	− 0.216	0.910*	0.930*	0.651	0.904*	0.670	0.910*
SIN	− 0.539	− 0.632	− 0.666	− 0.602	− 0.678	− 0.631	− 0.719	0.773	− 0.674	− 0.685	− 0.456	− 0.661	− 0.387	− 0.690
GNP	− 0.569	− 0.665	− 0.700	− 0.633	− 0.716	− 0.670	− 0.759	0.734	− 0.709	− 0.721	− 0.479	− 0.696	− 0.419	− 0.724
Total	− 0.406	− 0.498	− 0.535	− 0.474	− 0.602	− 0.513	− 0.588	0.858	− 0.545	− 0.553	− 0.346	− 0.528	− 0.267	− 0.567

Gene name	UGT74C1		ST5c	ST5b										FMOGS-OX2
Paralogue	1	2		1	2	3	4	5	6	7	8	9	10	
AGSL														
GIB	0.971**	0.713	0.961**	0.866	0.926*	0.961**	0.947*	0.817	0.684	0.503	0.641	0.947*	0.820	0.924*
GRA	0.947*	0.837	0.867	0.886*	0.853	0.945*	0.926*	0.883*	0.612	0.456	0.628	0.991**	0.697	0.871
SIN	− 0.712	− 0.697	− 0.627	− 0.677	− 0.692	− 0.756	− 0.690	− 0.745	− 0.458	− 0.301	− 0.134	− 0.783	− 0.489	− 0.633
GNP	− 0.748	− 0.730	− 0.659	− 0.711	− 0.719	− 0.789	− 0.727	− 0.775	− 0.480	− 0.321	− 0.185	− 0.821	− 0.514	− 0.668
Total	− 0.586	− 0.625	− 0.493	− 0.568	− 0.578	− 0.640	− 0.567	− 0.658	− 0.366	− 0.226	0.008	− 0.671	− 0.370	− 0.508

Gene name	FMOGS-OX5	AOP2			GSL-OH			BZO1p		MYB28			MYB29	
Paralogue		1	2	3	1	2	3	1	2	1	2	3	1	2
AGSL														
GIB	0.940*	0.766	0.128	− 0.626	0.641	0.948*	0.641	0.944*	0.898*	− 0.914*	0.936*	0.949*	0.923*	0.942*
GRA	0.926*	0.881*	− 0.098	− 0.698	0.628	0.854	0.628	0.896*	0.919*	− 0.970**	0.943*	0.947*	0.859	0.901*
SIN	− 0.735	− 0.702	0.386	0.119	− 0.134	− 0.617	− 0.134	− 0.672	− 0.704	0.773	− 0.508	− 0.709	− 0.627	− 0.665
GNP	− 0.768	− 0.739	0.373	0.182	− 0.185	− 0.648	− 0.185	− 0.706	− 0.740	0.810	− 0.562	− 0.747	− 0.659	− 0.700
Total	− 0.621	− 0.618	0.471	− 0.028	0.008	− 0.484	0.008	− 0.548	− 0.593	0.667	− 0.354	− 0.587	− 0.501	− 0.540

Numbers below gene names indicate gene paralogues

*GIB* 3-methylsulphinylpropyl, *GRA* 4-methylsulfinylbutyl, *SIN* 2-propenyl/Allyl, *GNP* but-3-enyl

*^,^**Correlation is significant at the 0.05 and 0.01 level, respectively (2-tailed)

### Glucosinolate analysis

The GSL extraction was performed according to Hennig et al. (2012) with slight modifications, using 200 mg of fresh leaves in 1 mL 90% hot methanol, followed by one re-extraction in 1 mL of boiling 70% methanol. Extracts were subsequently analysed by means of high-performance liquid chromatography (HPLC) as described on a gradient system HPLC (Spectra Physics, San Jose, CA) using a Nova-Pak C18 column (150 mm) with a flow rate of 1 mL/min, injection volume of 20 µL and column temperature of 20 °C. Solvents and gradient were used as it was described by Hennig et al. (2012). GSL identification was based on retention times compared to the internal standard glucotropaeolin or sinigrin: glucotropeolin (25 µL, 3 mM) as internal standard for WT and transgenic plant analysis, while sinigrin (60 µL, 3 mM) was the standard GSL for the extracts from the cell culture experiments. AGSL quantification was calculated using the internal reference and relative response factors (RRFs) in µmol/g sample.

### Preparation of plant extracts and HT29 cell treatment

GSLs from 200 mg fresh weight (FW) of DH-WT1 and DH-TP6 plants were first extracted in 1 mL of boiling 90% methanol for 10 min, keeping the samples in a water bath at 75 °C and vortexing regularly. The supernatant was collected after centrifugation (5 min at 25,000×*g*), stored in ice and the pellet was re-extracted once more in the same way. Methanol was evaporated using a nitrogen evaporator (Tianjin Novelab Ltd., China). The dried content was diluted and took up to 1 mL serum-free Dulbecco´s modified Eagle medium (DMEM; Sigma-Aldrich, Gillingham, UK) giving a stock solution of 200 mg FW/mL, which was frozen in aliquots of 200 μL each until samples were added to the cells.

HT29 human colon cancer cells (Cohen et al. 1999) were cultured in DMEM, supplemented with 10% (v/v) fetal bovine serum and penicillin (50 IU/mL)/streptomycin (50 µg/mL) in a humidified 5% CO_2_ incubator at 37 °C and seeded at a concentration of 1 × 10^6^ cells per well into 6-well culture dishes (CELLLSTAR® 6 Well Cell Culture Multiwell Plates, Greiner Bio One) 24 h prior to incubation with the plant extracts. In order to test the chemoprotective potential on colon cancer cells, these were incubated with different concentrations of plant extracts for 24 h. The plant extracts were derived from the wild type as well as from a *35S::BoMYB29* transgenic plant corresponding to 10, 100 and 1000 μg of plant material (wet weight) per mL of cell culture medium, concentrations suggested by Gill et al. (2004) and Boyd et al. (2006). A control of incubated colon cancer cells grown in serum-free DMEM was run in parallel. After 24 h, the incubated colon cells were harvested by trypsination, re-suspended in DMEM and centrifuged at 300×*g* for 8 min at RT. Subsequently, the cell pellet was re-suspended in serum-free DMEM and cell number and viability of the treated cells were assessed by Trypan Blue assay. The cell concentration was adjusted to 1.5 × 10^6^ cells/mL and duplicated samples were either exposed to 75 μM H_2_O_2_ (induction of DNA damage) or serum-free DMEM (control) for 5 min on ice. The reaction was stopped by centrifuging cells at 280×*g* 5 min at 4 °C and washing the cell pellet once with serum-free DMEM. All cell culture reagents were provided by Lonza (Basel, Switzerland).

### Single cell gel electrophoresis: comet assay

A ‘Comet’ assay was performed to measure the resulting DNA damage. Alkaline single cell gel electrophoresis was conducted as described by Singh et al. (1988). In brief, treated HT29 cells were centrifuged (280×*g* 5 min at 4 °C) and the cell pellet was dissolved in low melting agarose before being transferred onto microscope slides. Slides were placed in lysis solution (100 mM Na_2_EDTA, 1% Triton X 100, 2.5 M NaCl, 10 mM Tris, pH 10) for 60 min at 4 °C and then transferred into the electrophoresis chamber containing ice-cold buffer (1 mM Na_2_EDTA, 300 mM NaOH, pH ≥ 13) and left for 20 min to allow the DNA to unwind. Afterwards, electrophoresis was performed for 20 min at 1.25 V/cm and 300 mA at 4 °C. Neutralisation buffer (0.4 M Tris, pH 7.5) was used to wash slides prior to staining with ethidium bromide (Sigma-Aldrich). Microscopic images of tail migration of DNA from the central cell mass were analysed using the Komet 5.5 software (Kinetic Imaging, Kinetic Imaging, Liverpool, UK), which calculated the proportion and extent of DNA migration. Fifty cells were analysed per slide. After incubation with the different plant extracts and concentrations, three independent experiments (one per sample) with three replicates each were performed. All the chemicals for single cell gel electrophoresis were purchased from Sigma-Aldrich.

The data from the present experiment were statistically evaluated using ANOVA for repeated measures with Tukey’s post hoc test.

### Data availability

All data generated from the Affymetrix Brassica Exon 1.0 ST Array analysis have been deposited in the NCBI Gene Expression Omnibus (Edgar et al. 2002) and are accessible through GEO Series, accession number GSE39951 (http://www.ncbi.nlm.nih.gov/geo/query/acc.cgi?acc=GSE39951).

## Results

### Aliphatic glucosinolate production in *B. oleracea* genotypes correlates with the expression of glucosinolate biosynthesis genes

**Fig. 1:**
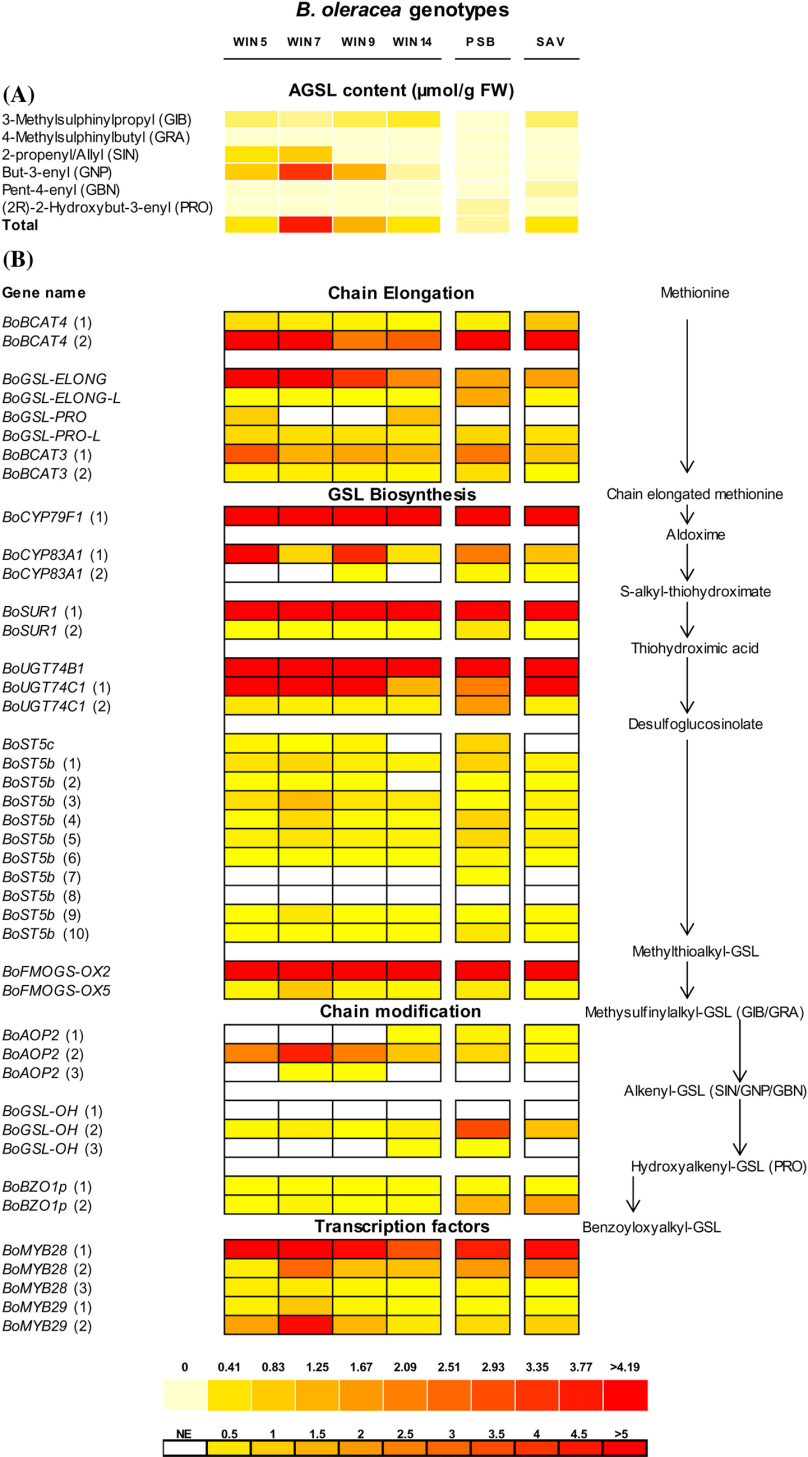
Heatmap of gene expression related to aliphatic glucosinolate (AGSL) biosynthesis and AGSL concentrations in leaves of *B. oleracea* wild accession Winspit (WIN), purple sprouting broccoli ‘Santee’ (PSB) and ‘Wintessa’ Savoy cabbage (SAV). Real-time quantitative qPCR analyses of the AGSL biosynthesis genes in four WIN plants (WIN5, 7, 9 and 14) and the F1 hybrid cultivars PSB and SAV (**a**). Expression levels are normalized to the expression of reference gene *BoGAPDH*. Aliphatic GSL concentrations in the same material (**b**). The boxed heatmap legend corresponds to gene expression, the boxless legend corresponds to the GSL concentrations. On the right side of the figure, the aliphatic GSL pathway is displayed and genes are arranged according to their role in the pathway. Full gene names and primers are listed in Supplemental Table S1. *NE* not expressed, *GSL* glucosinolate, *FW* fresh weight

In order to evaluate whether the content of AGSLs in the different genotypes analysed was correlated to gene expression, the four Winspit plants plus the PSB and SAV cultivars were used to perform HPLC analyses, microarray and qRT-PCR transcription profiling assays. Profiles and quantities of AGSLs differed between leaves of Winspit and the cultivated PSB and SAV lines. Although variation was observed among Winspit individuals, in general the wild genotypes displayed a higher AGSL concentration compared to the two crops, particularly for the alkenyl-GSLs gluconapin (GNP) and sinigrin (SIN) (Fig. 1a, Table S3). In particular, WIN7 showed the highest concentration of GNP and SIN compared to all other genotypes. The striking differences in GSL levels among the examined plant material might be due to differences in either expression or function of genes involved in GSL biosynthesis. To determine gene expression, leaf material of all genotypes was used for Affymetrix Brassica Exon 1.0 ST microarray hybridisation. The results of the microarray assay were used to perform a Principle Component Analysis (PCA) (Fig. S1), showing that the three *B. oleracea* types differed from each other with respect to overall gene expression. As expected, WIN plants were more heterogeneous than both the F1 hybrid cultivars and, on the whole, more similar to SAV than to PSB plants. When the combined WIN expression data were compared to the two cultivars, 656 probe sets were significantly differentially expressed between WIN and PSB and 699 probe sets between WIN and SAV. In total, 170 probe sets were significantly differentially expressed between WIN and both PSB and SAV (Table S4). Gene Ontology analysis of these differentially expressed genes revealed that GO terms related to ‘mucilage metabolism and extrusion’, ‘seed coat development’ and ‘seedling development’ were significantly overrepresented in the WIN genotypes (Table S5).

In order to specifically analyse genes of the GLS pathways, a panel of 216 probe sets, representing genes that were previously considered to be relevant for GSL biosynthesis (Wang et al. 2011), was examined for differential expression between WIN plants and the two cultivars (Fig. S2). Four GSL genes were significantly differentially expressed between WIN and PSB: *BoGSL*-*OH* (Bra021670, predicted to encode a 2-oxoacid dependent dioxygenase) and *BoIPMI SSU2* (Bra004744, predicted to encode an isopropylmalate dehydratase) were both down-regulated in WIN, while *BoGSTF9* (Bra022815, predicted to encode glutathione S-transferase phi9) and *BoST5a* (Bra008132, predicted to encode desulfoglucosinolate sulfotransferase 5a) were both up-regulated in WIN. The same genes, except for *BoGSTF9*, were differentially expressed between WIN and SAV.

The number of genes differentially expressed between each Winspit line and the cultivars varies. Three probe sets (rres089630.v1_st, Bra023450; rres017223.v1_st, Bra021670; rres045980.v1_x_st, Bra021670) were found to be consistently differentially expressed between WIN7 and the other genotypes. Of these genes, Bra023450 was up-regulated (3.5 vs. SAV, 5.2 vs. PSB), while Bra021670 was down-regulated. Similar results were obtained for WIN5 (three probe sets) and WIN9 (five probe sets). Of these probe sets, two were consistently differentially expressed in all WIN lines compared to the cultivated plants: Bra023450 (rres089630.v1_st) encoding a 3-methyladenine DNA glycosylase and Bra021670 (rres017223.v1_st) encoding a putative dioxygenase, were up-regulated and down-regulated in WIN, respectively.

To complement the microarray analysis and to study the *B. oleracea* AGSL pathway in more detail, expression of different AGSL genes identified in *B. rapa* and *B. oleracea* (Li and Quiros 2002; Gao et al. 2006; Wang et al. 2011) was determined by qRT-PCR analysis. In addition, expression of the different paralogues of those genes, which may not have been distinguished by microarray analysis, was assessed. Altogether, the expression of 42 AGSL biosynthesis pathway genes was assessed (Fig. 1b). The difference in AGSL production in the *B. oleracea* genotypes used in this study showed a positive correlation with the expression of *BoMYB29* and *BoMYB28* transcription factor genes and *BoBCAT4*(1), *BoGSL*-*PRO*, *BoCYP79F1*, *BoSUR1*, *BoST5b*(3), *BoFMOGS*-*OX*(5) and *BoAOP2*(2) structural genes (Table 1). The high AGSL containing genotype, WIN7 showed the highest relative expression of these genes. In contrast, both cultivars and WIN14, which displayed low AGSL production, also showed low expression of the above-mentioned genes.

### *MYB29* from Winspit regulates the expression of several genes in *B. oleracea*

Although the AGSL concentrations in the WIN genotypes were naturally high, we wanted to investigate if overexpression of one of the transcriptional regulators of the AGSL pathway could increase the AGSL leaf content to even higher levels. Among the key regulators whose expression was positively and significantly correlated with AGSL content, we chose *BoMYB29*(2) since its expression in the different genotypes used in this study showed the same trend as the expression of *BoAOP2* (Fig. 1), a gene crucial for the synthesis of Alkenyl-GSL. Therefore, we cloned the WIN7 *MYB29*(2) gene (*BoMYB29*) by RT-PCR from RNA and constructed an *A. tumefaciens* vector under the control of the constitutive CaMV 35S promoter. The DH AG1012 genotype, which can be easily transformed (Sparrow et al. 2006), and plants of the wild *B. oleracea* accession Winspit, were genetically modified using the *Pro35S::BoMYB29* construct. Six and two independent transformants were obtained from DH AG1012 and from Winspit, respectively. Preliminary phenotypic data did not show any difference in AGSL type and concentration between WT and EV plants. Therefore, WT plants growing at the same environmental conditions as the *BoMYB29* transgenic lines were used as a control of the transformation.

In order to evaluate the effect of *BoMYB29*(2) overexpression, we used the same Affymetrix Brassica Exon 1.0 ST microarray as above. The expression levels of GSL biosynthesis related genes, consisting of 216 probe sets, representing 86 *Brassica* genes (Wang et al. 2011), were assessed in the transformed DH and Winspit lines and compared to the WT plants. A profile plot and a hierarchical clustering showed that many GSL related genes are up-regulated and several other genes down-regulated in the DH *BoMYB29*-overexpressing lines, compared to the control plants (Fig. S3a, b). Out of the 216 GSL related probe sets, a group of 46, representing 24 genes, was consistently up-regulated in the transgenic lines DH3, DH5 and DH6 (Fig. S3b; Tables S6, S7). The same analysis was performed for the two transgenic Winspit lines, compared to the control plants. Hierarchical clustering analysis of the Winspit *BoMYB29*-overexpressing lines demonstrated that a smaller number of genes was up-regulated as compared to the DH *BoMYB29* transgenic lines (Fig. S3c; Table S8). Only eleven probe sets (representing nine genes) were differentially expressed in W1, twenty probe sets in W2 (representing 16 genes), and eight probe sets (representing seven genes) were consistently differentially expressed in the two Winspit transgenic lines compared to the wild type.

**Fig. 2:**
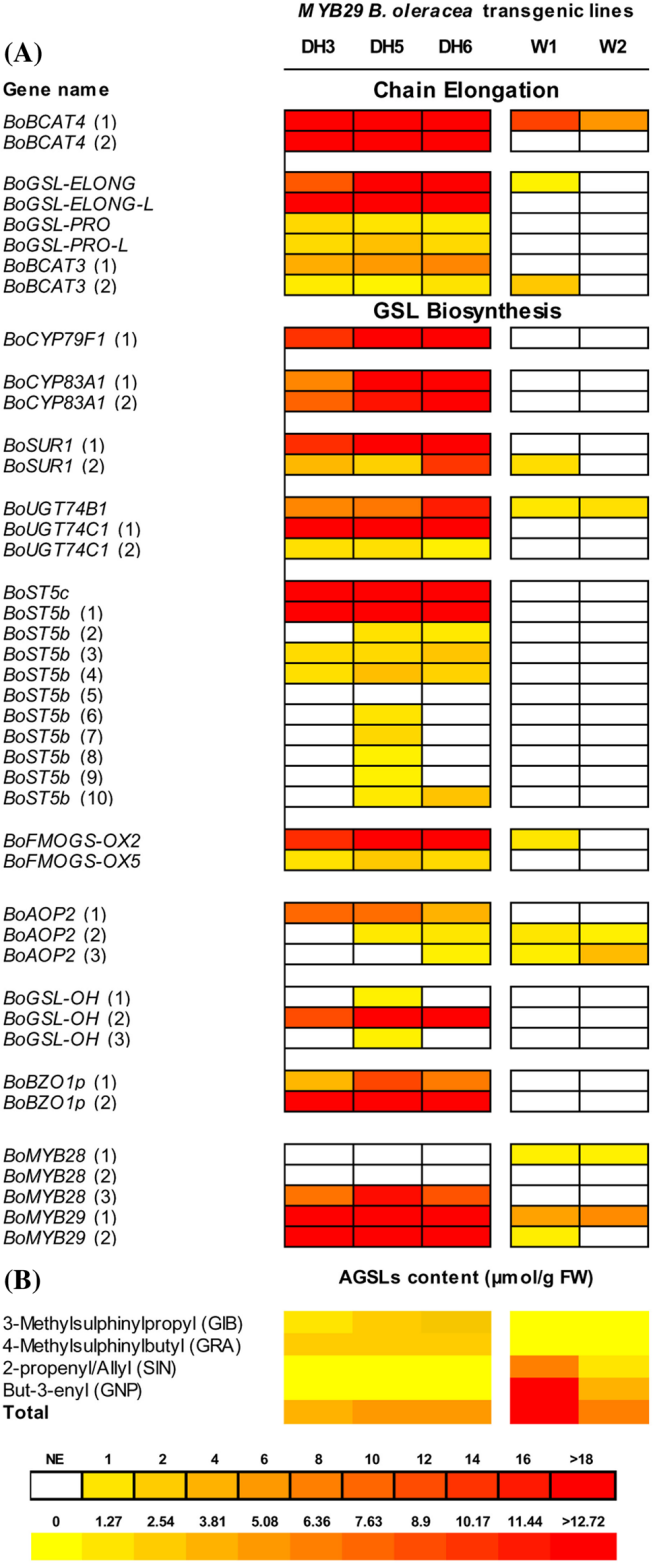
AGSL gene expression level and AGSL concentration in the *MYB29* overexpressing lines. Relative expression level of the 42 AGSL related genes in the *MYB29* DH AG1012 transgenic DH3, DH5 and DH6 lines compared to the wild type DH-WT1, and the *MYB29* Winspit overexpressing W1 and W2 lines compare to the wild-type W–WT1 (**a**). AGSL content in the *MYB29* overexpressing DH3, DH5, DH6 and W1, W2 lines compare to wild-type DH-WT1, W–WT2 plants respectively (**b**). Colours indicate expression level compared to *BoGAPDH* housekeeping gene expression. *NE* not expressed gene, *GSL* glucosinolate, *FW* fresh weight

Quantitative qRT-PCR experiments were performed to confirm the microarray data. Expression of the 42 AGSL genes analysed previously was studied for the *BoMYB29* transgenic lines and their respective WT backgrounds. *BoMYB29* was highly up-regulated in all transformants, especially in the DH lines (Fig. 2a). For the DH AG1012 *BoMYB29* overexpression lines, at least one paralogue of each of the 42 genes analysed was up-regulated in the transgenics compared to the WT, while *BoST5b* (gene 5) and *BoMYB28* (genes 1 and 2) were the only genes that did not display an up-regulation in the transgenic lines. *BoBCAT4* (genes 1 and 2), *BoGSL*-*ELONG* and *BoST5c* were highly up-regulated (Fig. 2a). Other genes with > 20-fold up-regulation in DH AG1012 transgenic lines were *BoCYP79F1*(1), *BoCYP83A1*(2), *BoSUR1*(1), *BoUGT74C1*(1), *BoST5B*(1), *BoFMOGS*-*OX2*, *BoGSL*-*OH*(2), *BoBZO1p*(2). Winspit overexpressing lines displayed less activity of the transgene compared to the DH tranformants. In fact, in the Winspit transgenic plants just a few GSL genes displayed an up-regulation due to the overexpression of *MYB29*. Among the genes highly expressed in the Winspit overexpressing lines are *BoBCAT4*(1), *BoBCAT3*(2) and *BoSUR1*(2) with more than two times up-regulation in W1 compared to the respective WT. In general, *BoBCAT4* and *BoBAT5*, encoding the sodium symporter protein 5, were the strongest expressed genes among the up-regulated ones in the overexpressing lines.

### *BoMYB29* overexpression increases the AGSL production in *B. oleracea* plants

The overexpression of *BoMYB29* did not only induce the overexpression of several GSL related genes, but also led to higher levels of AGSLs in the transgenic *B. oleracea* plants, when compared to their WT control plants (Figs. 2b, S4; Table S3). As for the DH AG1012 *BoMYB29* transgenics, a higher production of methylsulphinylalkyl-GSLs compared to the WT plants was observed. The expression of several genes showed a significant correlation (p < 0.01) with the production of methylsulphinylalkyl-GSLs (Table 2). For instance, the expression of *BoBCAT4*(2), *BoGSL*-*Elong*, *BoSUR1*(1), *BoUGT74B1*, *BoUGT74C1*(1) and *BoST5c* genes was significantly correlated with the production of glucoraphanin in the overexpressing plants, while the expression of *BoBCAT3*(1), *BoCYP83A1*(2), *BoST5b*(3) was correlated with both glucoraphanin and glucoiberin production.

On the other hand, in the *BoMYB29* overexpressing Winspit lines, high levels of alkenyl-GSLs were found, while no methylsulphinylalkyl-GSLs could be detected, suggesting that all the methylsulphinylalkyl-GSLs were converted into alkenyl-GSLs (Fig. 2b). The two Winspit overexpressing lines contained significantly more 2-propenyl/allyl (sinigrin) and butenyl GSL (gluconapin), as well as total AGSL levels, than their control. The total GSL content was positively and significantly correlated with the expression of the *BoBCAT3*(2) gene in Winspit transgenic lines (Table 2). Conversely, DH AG1012 plants produced methylsulphinylalkyl-GSLs and no alkenyl-GSL form (Fig. S4). Amplification and sequencing of the DH AG1012 *BoGS*-*ALK* (*BoAOP2*) gene, encoding the 2-oxoglutarate-dependent dioxygenase involved in this biochemical conversion, showed the presence of a 2-bp deletion in exon 2 of *BoAOP2* paralogues, as it was previously shown by Gao et al. (2004) in broccoli.

### DH6 GSL extract produces a protective effect against H_2_O_2_-induced DNA damage

**Fig. 3:**
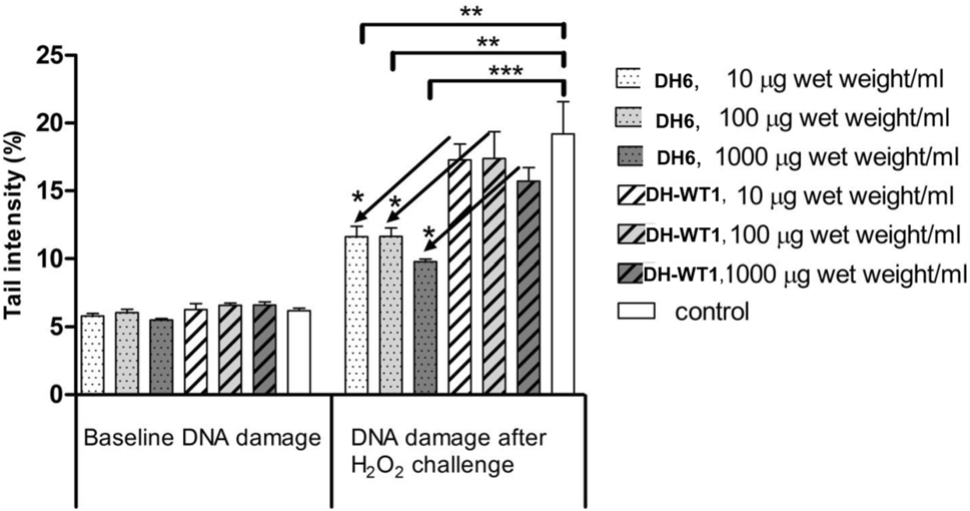
Chemopreventive effect of GSL-rich extract from DH AG1012 *BoMYB29* transgenic plant leaves against H_2_O_2_ induced DNA damage. Comparison of the plant extracts against untreated control (white bars) without H_2_O_2_ (baseline DNA damage) and with H_2_O_2_ (DNA damage after H_2_O_2_ challenge). Comparison between DH6 plant treatments and DH-WT1 plant treatments: DH6, 1000 versus DH-WT1, 1000; DH6, 100 versus DH-WT1, 100; and DH6, 10 versus DH-WT1, 10, without H_2_O_2_ (left part) and with H_2_O_2_ (right part). DH6: DH AG1012 transgenic line 6; DH-WT1: DH AG1012 wild type. *p < 0.05; **p < 0.01; ***p < 0.001 (ANOVA for repeated measures with Tukey’s post hoc test)

The chemopreventive properties of *B. oleracea* plant extracts were tested by assessing their potential to protect human colon HT29 cells from DNA damage by hydrogen peroxide, a proven genotoxic compound. For this assay, we used the wild type DH-WT1 and the transgenic line DH6, showing the highest GSL content among the obtained transformants. The Comet assay (single-cell gel electrophoresis) is a simple method for measuring DNA strand breaks in eukaryotic cells (Collins 2004). Comet assay experiments performed without addition of hydrogen peroxide showed that the plant extracts did not induce DNA damage themselves as there was no difference in baseline DNA damage after 24 h incubation with plant extracts compared to incubation with serum-free medium (control) (Repeated Measures ANOVA with Tukey’s post hoc test, p = 0.074) (Fig. 3). Incubating the control sample with hydrogen peroxide led to the induction of considerable DNA damage (Tail fluorescence intensity: 19.2 ± 4.1% mean ± SD Tail DNA) (Fig. 3). Analysis with Repeated Measures ANOVA revealed that results for hydrogen peroxide induced DNA damage differed significantly according to treatment (p = 0.0002). Tukey’s post hoc test showed that while incubation for 24 h with wild-type extracts had no effect on DNA strand breaks induced by hydrogen peroxide, incubation with DH6 and DH-WT1 GSL extracts significantly reduced DNA damage compared to the control sample (p < 0.01, p < 0.01 and p < 0.001 for 10 µg/mL, 100 µg/mL and 1000 µg/mL fresh weight of transgenic plant extracts compared to serum-free medium, respectively). Extracts from the DH6 transgenic line were also significantly more effective in preventing DNA strand breaks than the DH-WT1 extracts at the respective concentrations (p < 0.05 for 10 µg/mL DH6 vs. DH-WT1, 100 µg/mL DH6 vs. DH-WT1 and 1000 µg/mL DH6 vs. DH-WT1).

## Discussion

The focus of this study was to analyse the expression of the genes involved in the AGSL biosynthesis in *B. oleracea*, and establish a correlation between AGSL production and gene expression in WIN plants, and PSB and SAV varieties.

The AGSL pathway is well known in *B. rapa*, where a comprehensive map of the involved genes has been created (Zang et al. 2009; Wang et al. 2011). Moreover, *B. oleracea* genome is already available (Liu et al. 2014), showing a genetic similarity with the *B. rapa* genome and some knowledge regarding the *B. oleracea* AGSL pathway has been also achieved (Yi et al. 2015). In this study, the expression of most of the *B. oleracea* genes homologous to the previously identified *B. rapa* genes was assessed. However, for the *BoST5b* and *BoGSL*-*OH* genes, one paralogue identified in *B. rapa* was not found in *B. oleracea*. Our microarray data showed also that *BoGSL*-*OH*, a *2*-*oxoacid*-*dependent dioxygenase*, is down-regulated in WIN compared to the cultivated genotypes. The gene encoding *Bo*GSL-OH is necessary for the synthesis of the (2R)-2 Hydroxybut-3-enyl-GSL (progoitrin) from But-3-enyl GSL (gluconapin). Therefore, this could be the main reason why the alkenyl-GSLs, in particular sinigrin and gluconapin, are predominant in the wild genotype.

Moreover, our study showed a significant positive correlation between the relative expression levels of the *BoMYB28* and *BoMYB29* transcription factor genes with the AGSL content. Additionally, several structural genes were also positively correlated with AGSL production. It is therefore highly likely that expression of the *BoBCAT4*, *BoGSL*-*PRO*, *BoCYP79F1*, *BoSUR1*, *BoST5b*, *BoFMOGS*-*OX5* and *BoAOP2* genes is controlled by the BoMYB28 and/or BoMYB29 transcription factors. Moreover, the high, positive correlation between the expression of the two *MYB* genes and *BoAOP2* with the concentration of 2-propenyl (sinigrin) and but-3-enyl (gluconapin) AGSLs highlights the important role of *BoMYB29* and *BoMYB28* in the regulation of *BoAOP2* and the synthesis of alkenyl-GSLs in *B. oleracea*. Such control of AGSL biosynthesis gene expression is in agreement with what has been found for *A. thaliana*, where *AtMYB28* and *AtMYB29* also regulate the expression of *AtAOP2*, among other genes (Sønderby et al. 2007; Hirai et al. 2007). Additionally, *AtAOP2* is responsible for the conversion of the methylsulfinylalkyl-GSLs into the alkenyl form, which is essential for the production of alkenyl-GSLs (Neal et al. 2010). On the other hand, expression of *Brassica AOP2* genes has been studied before, for both *B. rapa* (Wang et al. 2011; Zhang et al. 2015) and *B. oleracea* (Gao et al. 2004; Li et al. 2014), as well as one of its regulatory genes, *MYB28,* which in *B. rapa* has been identified as negative regulator of *BrAOP2* (Seo et al. 2016). In this work, expression analysis of GSL genes in *B. oleracea* plants showed a positive correlation between *BoMYB29* and *BoAOP2* gene expression, especially with the *BoAOP2(2)*.

Since the expression of *BoMYB29* was positively correlated with that of *BoAOP2*, we performed functional analysis of *MYB29* in two *B. oleracea* genotypes (Winspit and DH AG1012) with different GSL profiles, and analysed the effects of overexpression of the *MYB29* gene, at the transcriptional and phenotypic level. In the model species *A. thaliana*, closely related to *B. oleracea*, knockout mutant and ectopic expression studies have demonstrated the positive regulation that *MYB28* and *MYB29* transcription factors play in the AGSL pathway (Hirai et al. 2007; Beekwilder et al. 2008; Gigolashvili et al. 2008; Yin et al. 2017). However, while *MYB28* has been identified as the dominant regulator, *MYB29* is suggested to have a minor rheostat role in constitutive glucosinolates biosynthesis in *Brassica* (Gigolashvili et al. 2007; Hirai et al. 2007; Sønderby et al. 2007, 2010). Other studies found an important activation role of *AtMYB29* for the AGSL biosynthesis and suggested that *AtMYB29* contributes equally together with *AtMYB28* to regulate the AGSL biosynthesis in *A. thaliana* (Beekwilder et al. 2008). Furthermore, when a *B. oleracea BoMYB29* gene isolated from kale (*B. oleracea* var. *acephala*) was overexpressed in *A. thaliana,* it enhanced the expression of AGSL biosynthetic genes and increased the accumulation of methylsulphinyl GSLs including glucoraphanin significantly (Araki et al. 2013). Therefore, our interest was focused in evaluating the effect of *BoMYB29* on AGSL production in *B. oleracea*. To our knowledge, this is the first study showing a successful overexpression of *MYB29* in *B. oleracea*.

In agreement with our observations in the *BoMYB29* DH AG1012 overexpression lines, the *BoMYB29* overexpression in *myb28myb29* *Arabidopsis* mutant, the methylsulphinyl GSL content, including glucoraphanin was greatly increased, indicating the suitability of BoMYB29 as a regulator for increasing methylsulphinyl GSL content. (Araki et al. 2013). Unfortunately, the analysis of plants overexpressing *BoMYB29* and potential *AtMYB29* knock-out mutants was not conclusive as to detect the direct transcriptional targets of *AtMYB29* in *A. thaliana* (Sønderby et al. 2010; Araki et al. 2013).

Our results also demonstrate that *BoMYB29* plays a key role in the production of AGSLs in *B. oleracea,* activating all the genes in the pathway. The *BoMYB29* overexpressing lines created for this study displayed a higher expression of the *B. oleracea* orthologues of *A. thaliana* genes previously found to be up-regulated by this transcription factor in *A. thaliana* (Gigolashvili et al. 2008). Our microarray data show the same mode of action for *B. oleracea.* The analysis of the whole AGSL pathway in *B. oleracea*, allows us to affirm that the *B. oleracea* gene encoding the *BILE ACID TRANSPORTER 5* (*BoBAT5*) is the most important target gene regulated by *BoMYB29*. The *AtBAT5* gene has been identified in *A. thaliana* to encode a plastic transporter involved in the AGSL biosynthesis and to be a target of *AtMYB29* (Gigolashvili et al. 2009; Sawada et al. 2009).

On a phenotypic level, the overexpression of *BoMYB29* increased the production of aliphatic glucosinolates in both modified *B. oleracea* genotypes, Winspit and DH AG1012. *BoMYB29*-DH AG1012 lines showed a higher content of 3-methylsulfinylpropyl GSL (GIB) and 4-methylsulfinylbutyl GSL (GRA) compared to the untransformed (WT) plants. On the other hand, the Winspit overexpressing lines, which have a functional *BoAOP2* gene, produced a significantly higher content of 2-propenyl/Allyl (SIN) and But-3-enyl (GNP). Similar studies have been performed in *A. thaliana* where the overexpression of *AtMYB29* increased the levels of short and long—chained aliphatic glucosinolates including GIB and GRA (Gigolashvili et al. 2009). This confirms that MYB29 is a key transcription factor, which regulates the production of aliphatic GSLs in *B. oleracea* plants. The absence of Alkenyl GSLs in the DH AG1012, even when *BoMYB29* is overexpressed, can be ascribed to the non-functionality of AOP2, which is the enzyme responsible for the production of Alkenyl GSLs from Methylsulfinylalkyl GSLs. The absence of alkenyl GSLs in the DH AG1012 transgenic lines as well as in the DH AG1012 WT plants is fully in line with the absence of one or more steps in the AGSL biosynthetic pathway in this genotype, blocking the biosynthesis of alkenyl-GSLs. The gene is also not functional in *B. oleracea* var. *italica* (Gao et al. 2004), which is part of the ancestry of DH AG1012 (Sparrow et al. 2006). Instead of Alkenyl GSLs, the DH accumulates methylsulphinylalkyl-GSLs, including glucoraphanin, contributing to the anti-cancer properties of *B. oleracea* (Juge et al. 2007; Chi et al. 2015).

In our assays, a concentration of 1000 µg/mL of DH6 plant extract displayed the highest HT29 cancer cell protective effect against subsequent DNA damage induced by hydrogen peroxide. The cell protection observed in our experiment may be produced by GSL degradation products (ITCs), which have a widely documented chemopreventive activity (Traka and Mithen 2009). DNA damage protective effect might be also produced by the complex mixture of compounds present in the plant extract, including the non-volatile hydrolysis products of the GSLs identified and a range of phenolic compounds, as it was suggested by Boyd et al. (2006) from observations using watercress (*Nasturtium officinale*) extracts.

Glucosinolates are also useful in agricultural applications. Glucosinolate profile and content have been assessed in the wild *B. oleracea* Winspit showing high levels of alkenyl-GSL, in the leaves which make it particularly interesting for biofumigation applications (Gols et al. 2008; Zuluaga et al. 2015). Our expression analysis contribute to a better understanding of the mechanism that regulates the biofumigation properties of Winspit, suggesting that several genes involved in the GSL pathway play a key role in the biofumigation potential of the wild genotype. The high AGSL production in WIN7 and in the Winspit *BoMYB29* overexpressing plants was mainly due to the high presence of the 3-butenyl GSL (gluconapin) and the 2 propenyl/Allyl (sinigrin). These alkenyl-GSLs are produced from the methylsulphinylalkyl-GSLs by the 2-oxoglutarate-dependent dioxygenase encoded by the *AOP2* gene. Therefore, our results suggest that the higher production of sinigrin in the Winspit plants is due mainly to the presence of a functional *AOP2* gene, which is regulated by *MYB29* in *B. oleracea*. Therefore, *BoAOP2* gene and the gene encoding *BoMYB29* transcription factors could be targeted in breeding programmes aimed at improving the biofumigation properties in *Brassicas*.

In conclusion, overexpression of the *BoMYB29* gene in *B. oleracea* plants up-regulates the AGSL pathway and increases the production of methylsulphinylalkyl GSLs such as the anti-cancer metabolite, glucoraphanin, or alkenyl glucosinolates including the toxic and pungent sinigrin. This difference largely depends on the presence or absence of a functional *BoAOP2* allele in a given genotype. Therefore, the results presented in this study illustrate how the *BoMYB29* gene from a wild *B. oleracea* can be used by plant breeders towards improving agricultural practices, by developing *B. oleracea* species with higher allelopathic properties against pathogens and pests. At the same time, increasing the synthesis of this transcription factor in particular genotypes, including those derived from economically important crops, improves anticancer effects and thus beneficial properties of *Brassica* plants for human health.

## Electronic supplementary material

Below is the link to the electronic supplementary material.

Supplementary material 1 (XLSX 21 kb)

Supplementary material 2 (DOC 78 kb)

Supplementary material 3 (XLSX 74 kb)

Supplementary material 4 (XLSX 58 kb)

Supplementary material 5 (XLSX 108 kb)

Supplementary material 6 (XLSX 11 kb)

Supplementary material 7 (DOCX 79 kb)

Supplementary material 8 (DOCX 96 kb)

Supplementary material 9 (XLSX 35 kb)

Supplementary material 10 (XLSX 16 kb)

Supplementary material 11 (XLSX 43 kb)

Supplementary material 12 (DOCX 272 kb)
