# Supplementary material for: Overexpression of the MYB29 transcription factor affects aliphatic glucosinolate synthesis in Brassica oleracea
Source: Plant Mol Biol. 2019 Jun 12;101(1):65–79. doi: 10.1007/s11103-019-00890-2 (PMC6695347; doi:10.1007/s11103-019-00890-2)
Supplement: Supplementary file 2 — Supplementary material 2 (DOC 78 kb) [file 11103_2019_890_MOESM2_ESM.doc]

**Fig. S1. Principle component analysis (PCA) of the *B. oleracea* gene expression data as determined by Affymetrix Brassica Exon 1.0 ST Array hybridisation.** PCA analysis of Brassica Exon Array data from leaves of Savoy cabbage ‘Wintessa’ (SAV; blue triangles), purple sprouting broccoli ‘Santee’ (PSB; red squares) and of individual plants of the wild accession ‘Winspit’ (WIN5, grey; WIN7, pink; WIN 9, green; and WIN14, brown circles). For the two cultivars, twelve plants were divided in four groups of three plants each and analysed per group. For Winspit, leaf material from four individual plants was analysed separately.


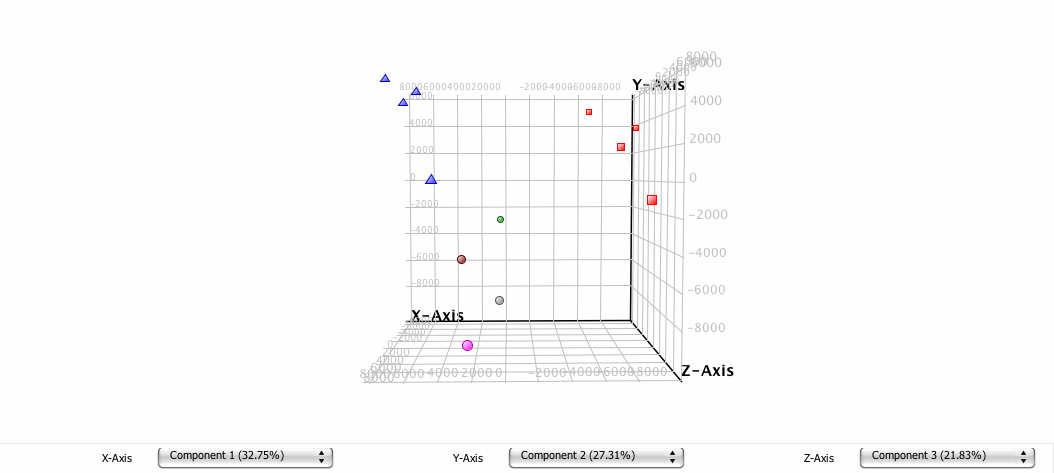


**Overexpression of the MYB29 transcription factor affects aliphatic glucosinolate synthesis in *Brassica oleracea***

Plant Molecular Biology

Diana L. Zuluaga^1,7*^, Neil S. Graham^2^, Annett Klinder^3^, A.E. Elaine van Ommen Kloeke^4^, Angelo R. Marcotrigiano^5^, Carol Wagstaff^3^, Ruud Verkerk^6^, Gabriella Sonnante^7^, Mark G.M. Aarts^1^

^1^Laboratory of Genetics, Wageningen University, Droevendaalsesteeg 1, 6708 PB, Wageningen, the Netherlands

^2^Plant and Crop Sciences Division, School of Biosciences, University of Nottingham, Sutton Bonington Campus, Loughborough, LE12 5RD, Leicestershire, UK

^3^Department of Food and Nutritional Sciences, University of Reading, PO Box 226, Whiteknights, Reading, RG6 6AP, UK

^4^Department of Ecological Science, Faculty of Earth and Life Sciences, VU University Amsterdam, De Boelelaan 1085, 1081 HV, Amsterdam, the Netherlands

^5^Department of Soil, Plant and Food Science, University of Bari ˝Aldo Moro˝, Bari, Italy

^6^Food Quality and Design, Wageningen University, P.O. Box 17, 6700AA, Wageningen, the Netherlands

^7^Institute of Biosciences and Bioresources, National Research Council, Via G. Amendola 165/A, 70126, Bari, Italy

* E-mail: diana.zuluaga@ibbr.cnr.it
