# Supplementary material for: Overexpression of the MYB29 transcription factor affects aliphatic glucosinolate synthesis in Brassica oleracea
Source: Plant Mol Biol. 2019 Jun 12;101(1):65–79. doi: 10.1007/s11103-019-00890-2 (PMC6695347; doi:10.1007/s11103-019-00890-2)
Supplement: Supplementary file 8 — Supplementary material 8 (DOCX 96 kb) [file 11103_2019_890_MOESM8_ESM.docx]

**Fig. S3.TIFF**

**Expression data from RNA hybridization of *B. oleracea* *BoMYB29* transgenic plants to the 216 probe set representing GSL genes in the Affymetrix Brassica Exon 1.0 ST array**. Normalized intensity values of DH AG1012 *BoMYB29* overexpressing lines (DH3, DH5, DH6) with the wild-type control plants DH-WT1, DH-WT2 and DH-WT3 (A). Hierarchical clustering of GSL genes in DH AG1012 transgenic lines DH3, DH5 and DH6, compared to the DH-WT plants (B). Cluster analysis of GSL genes in *BoMYB29* overexpressing Winspit transgenic lines W1 and W2, compared to the wild-type W-WT plants (C). Heat map colours reflect normalized expression values.


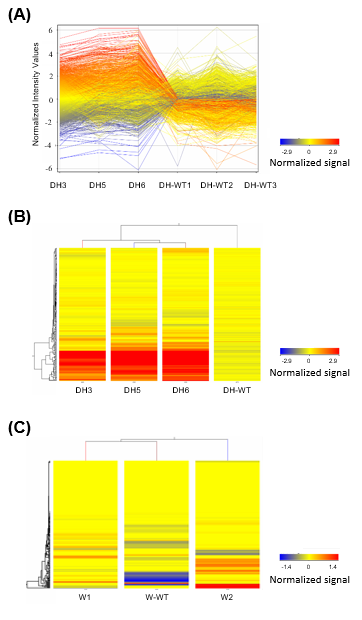


**Overexpression of the MYB29 transcription factor affects aliphatic glucosinolate synthesis in *Brassica oleracea***

Plant Molecular Biology

Diana L. Zuluaga^1,7*^, Neil S. Graham^2^, Annett Klinder^3^, A.E. Elaine van Ommen Kloeke^4^, Angelo R. Marcotrigiano^5^, Carol Wagstaff^3^, Ruud Verkerk^6^, Gabriella Sonnante^7^, Mark G.M. Aarts^1^

^1^Laboratory of Genetics, Wageningen University, Droevendaalsesteeg 1, 6708 PB, Wageningen, the Netherlands

^2^Plant and Crop Sciences Division, School of Biosciences, University of Nottingham, Sutton Bonington Campus, Loughborough, LE12 5RD, Leicestershire, UK

^3^Department of Food and Nutritional Sciences, University of Reading, PO Box 226, Whiteknights, Reading, RG6 6AP, UK

^4^Department of Ecological Science, Faculty of Earth and Life Sciences, VU University Amsterdam, De Boelelaan 1085, 1081 HV, Amsterdam, the Netherlands

^5^Department of Soil, Plant and Food Science, University of Bari ˝Aldo Moro˝, Bari, Italy

^6^Food Quality and Design, Wageningen University, P.O. Box 17, 6700AA, Wageningen, the Netherlands

^7^Institute of Biosciences and Bioresources, National Research Council, Via G. Amendola 165/A, 70126, Bari, Italy

* E-mail: diana.zuluaga@ibbr.cnr.it
